# The clinical pharmacology and potential therapeutic applications of 5‐methoxy‐N,N‐dimethyltryptamine (5‐MeO‐DMT)

**DOI:** 10.1111/jnc.15587

**Published:** 2022-03-08

**Authors:** Johannes T. Reckweg, Malin V. Uthaug, Attila Szabo, Alan K. Davis, Rafael Lancelotta, Natasha L. Mason, Johannes G. Ramaekers

**Affiliations:** ^1^ Faculty of Psychology and Neuroscience, Department of Neuropsychology and Psychopharmacology Maastricht University Maastricht The Netherlands; ^2^ Norwegian Centre for Mental Disorders Research (NORMENT), Institute of Clinical Medicine, University of Oslo, and Division of Mental Health and Addiction Oslo University Hospital Oslo Norway; ^3^ KG Jebsen Centre for Neurodevelopmental Disorders University of Oslo Oslo Norway; ^4^ Center for Psychedelic Drug Research and Education, College of Social Work The Ohio State University Columbus Ohio USA; ^5^ Center for Psychedelic and Consciousness Research, Department of Psychiatry Johns Hopkins School of Medicine Baltimore Maryland USA

**Keywords:** 5‐MeO‐DMT, clinical development, neuroinflammation, mental health

## Abstract

5‐methoxy‐N,N‐dimethyltryptamine (5‐MeO‐DMT) is a naturally occurring tryptamine that primarily acts as an agonist at the 5‐HT1A and 5‐HT2A receptors, whereby affinity for the 5‐HT1A subtype is highest. Subjective effects following 5‐MeO‐DMT administration include distortions in auditory and time perception, amplification of emotional states, and feelings of ego dissolution that usually are short‐lasting, depending on the route of administration. Individual dose escalation of 5‐MeO‐DMT reliably induces a “peak” experience, a state thought to be a core predictor of the therapeutic efficacy of psychedelics. Observational studies and surveys have suggested that single exposure to 5‐MeO‐DMT can cause rapid and sustained reductions in symptoms of depression, anxiety, and stress. 5‐MeO‐DMT also stimulates neuroendocrine function, immunoregulation, and anti‐inflammatory processes, which may contribute to changes in mental health outcomes. To date, only one clinical trial has been published on 5‐MeO‐DMT, demonstrating the safety of vaporized dosing up to 18 mg. Importantly, the rapid onset and short duration of the 5‐MeO‐DMT experience may render it more suitable for individual dose‐finding strategies compared with longer‐acting psychedelics. A range of biotech companies has shown an interest in the development of 5‐MeO‐DMT formulations for a range of medical indications, most notably depression. Commercial development will therefore be the most important resource for bringing 5‐MeO‐DMT to the clinic. However, fundamental research will also be needed to increase understanding of the neurophysiological and neural mechanisms that contribute to the potential clinical effects of 5‐MeO‐DMT and its sustainability and dissemination over time. Such studies are less likely to be conducted as part of drug development programs and are more likely to rely on independent, academic initiatives.

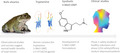

## Graphical abstract

5‐methoxy‐N,N‐dimethyltryptamine (5‐MeO‐DMT) is a naturally occurring tryptamine that primarily acts as an agonist at the 5‐HT1A and 5‐HT2A receptors, whereby affinity for the 5‐HT1A subtype is highest. Studies have suggested that single exposure to 5‐MeO‐DMT can cause rapid and sustained reductions in symptoms of depression, anxiety, and stress. 5‐MeO‐DMT also stimulates neuroendocrine function, immunoregulation, and anti‐inflammatory processes, which may contribute to changes in mental health outcomes. This review discusses the basis and potential for clinical application of 5‐MeO‐DMT formulations for a range of medical indications, most notably depression. Fundamental research will be needed to increase understanding of the neurophysiological and neural mechanisms that contribute to the potential clinical effects of 5‐MeO‐DMT and its sustainability and dissemination over time. Such studies are less likely to be conducted as part of drug development programs and are more likely to rely on independent, academic initiatives.

**Abbreviations**
5D‐ASC: 5‐dimensional altered states of consciousness
5‐HT: serotonin
5‐MeO‐DMT: 5‐methoxy‐N,N‐dimethyltryptamine
5‐MeOT: 5‐methoxytryptamine
5‐OH‐DMT: bufotenine
BDNF: brain‐derived neurotrophic factor
CYP2D6: cytochrome P450 2D6
CLRs: C‐type lectin receptors
DAMPs: damage‐associated molecular patterns
DMT: N,N‐dimethyltryptamine
EDI: ego dissolution inventory
IFN: interferon
IRF3/7: interferon ‐regulatory factor 3/7
IL‐1β: interleukin‐1β
IL‐6: interleukin‐6
IM: intramuscular injection
IUCN: International Union for Conservation of Nature
JNK: c‐Jun N‐terminal kinase
LSD: lysergic acid diethylamide
LH: luteinizing hormone
MAOI: monoamine oxidase inhibitor
MAO‐A: monoamine oxidase A
MDD: major depressive disorder
MAPKs: mitogen‐activated protein kinases
MEQ: the Mystical Experience Questionnaire
moDCs: monocyte‐derived dendritic cells
NF‐κB: nuclear factor kappa‐B
NOD: nucleotide‐binding oligomerization domain
NLRs: nucleotide‐binding oligomerization domain‐like receptors
PAMPS: pathogen‐associated molecular patterns
PES: peak experience scale
PRRs: pattern recognition receptors
PTSD: posttraumatic stress disorder
PFC: prefrontal cortex
RSN: resting‐state network
TNF‐α: tumor necrosis factor‐alpha
TLRs: toll‐like receptors

## 1 INTRODUCTION

5‐methoxy‐N,N‐dimethyltryptamine (5‐MeO‐DMT) is a naturally occurring tryptamine that can be found in seeds, bark, and leaves of a number of plants in the Amazonian rainforest (Pachter et al., 1959). Small amounts of 5‐MeO‐DMT have been traced in seeds of the *Virola* and *Anandenantera peregrine* (i.e., yopo, cohobo, and rapé) and in barks or leaves of plants such as *Dictyoloma incanescens* (Agurell et al., 1969; Pachter et al., 1959). Typically, such plant materials also contain other indole alkaloids such as N,N‐dimethyltryptamine (DMT) (Agurell et al., 1969; Pachter et al., 1959). 5‐MeO‐DMT is an entheogen of which indigenous use for shamanic purposes, tribal ceremonies, and healing rituals in South America and the Caribbean has been suggested (Weil & Davis, 1994), but strong evidence is lacking. There are also reports of the use of *Mucana pruriens* seeds that contain small amounts of indole alkaloids including 5‐MeO‐DMT (Bhattacharya et al., 1971; Szabo, 2003), as an aphrodisiac in Indian and Mexican cultures (Rätsch, 1998; Sathiyanarayanan & Arulmozhi, 2007; Sridhar & Bhat, 2007).

**FIGURE 1:**
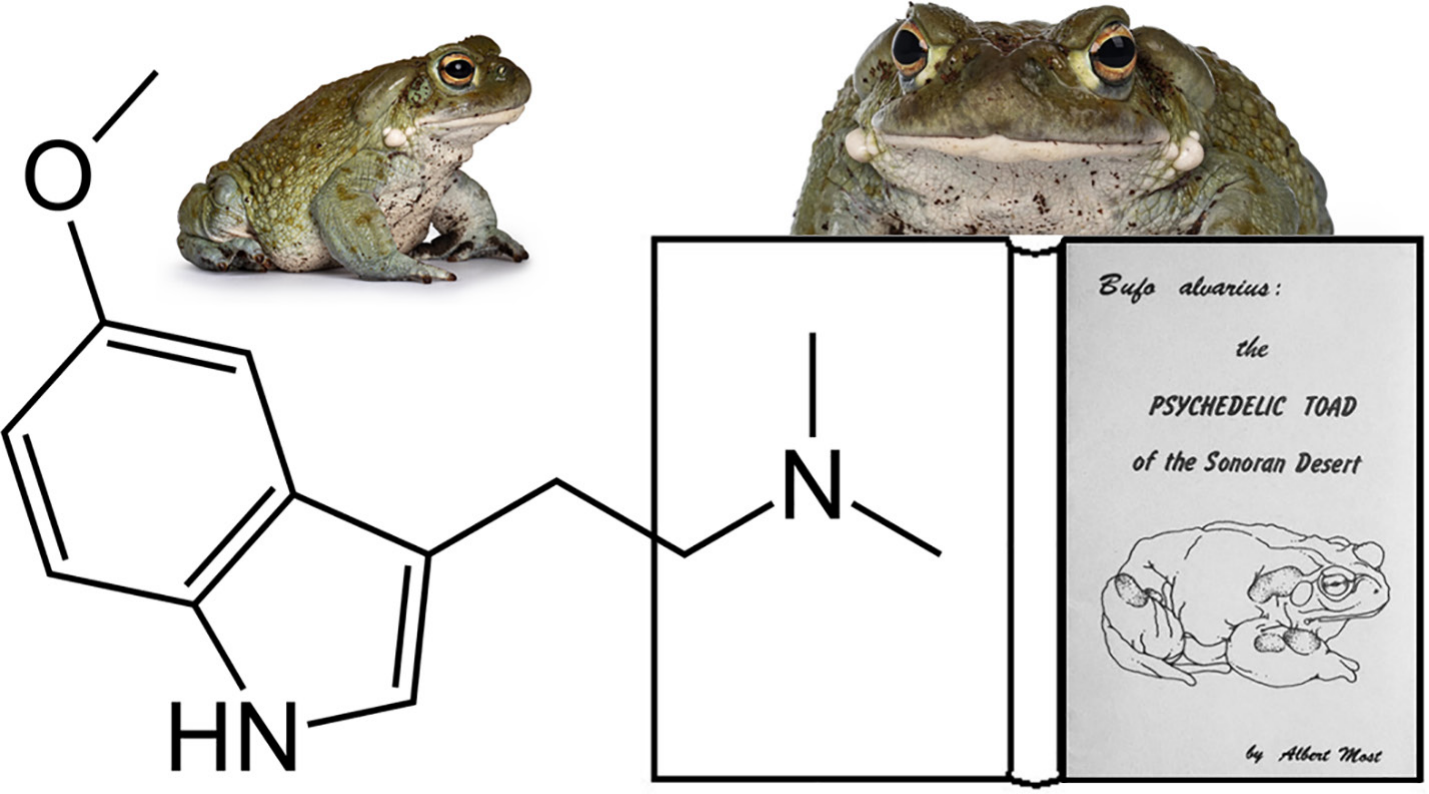
Depictions of the bufo alvarius toad, the structure of 5‐MeO‐DMT, and the cover of the pamphlet “Bufo alvarius: the psychedelic toad of the Sonoran desert” (Most, 1984), the first guide for collecting, drying, and smoking the toad venom

5‐MeO‐DMT has also been identified (Weil & Davis, 1994) as the primary psychoactive component of the parotoid gland venom of *Bufo alvarius* (*Incilius alvarius*), the Sonoran Desert toad, where it accounts for about 20–30% of its dry weight (Uthaug et al., 2019). The venom of the Bufo alvarius also contains bufotenine (Uthaug et al., 2019) from which 5‐MeO‐DMT is converted through O‐methyl transferase (Weil & Davis, 1994; Yu, 2008). The first published analysis of the venom of *Bufo alvarius* appeared in the 1960s (Chen & Kovarikova, 1967; Erspamer et al., 1965; Erspamer et al., 1967), which may have triggered experimentation with the toad’s venom in Western cultures. In 1984, an under‐ground pamphlet (see Figure 1) titled “Bufo Alvarius, the Psychedelic Toad of the Sonoran Desert” (Most, 1984) appeared. It was seemingly the first guide for collecting, drying, and smoking the venom of the Sonoran Desert toad. It created an opportunity for researchers and psychonauts (Weil & Davis, 1994), and its popularity as a psychedelic spread quickly (Davis et al., 2018).

It has been speculated that 5‐MeO‐DMT might also be endogenously produced in humans because its derivative bufotenine (5‐OH‐DMT) and its structural analog N,N‐dimethyltryptamine (DMT) have been detected in urine, blood, and cerebrospinal fluid (Christian et al., 1975; Corbett et al., 1978; Narasimhachari & Himwich, 1973; Sitaram et al., 1987; Smythies et al., 1979). Other studies have contradicted these findings however (Forsstrom et al., 2001; Huszka et al., 1976; Narasimhachari et al., 1974; Narasimhachari & Himwich, 1972) while a pooled analysis indicated that such markers were only present in limited fractions of study participants (Ermakova et al., 2021). One study also showed the presence of indoleamine precursors of 5‐MeO‐DMT in the pineal gland in rats (Miller & Maickel, 1970), while in vitro work on human pineal extract suggested that 5‐MeO‐DMT can be synthesized through further methylation of the precursor 5‐methoxytryptamine (5‐MeOT) or bufotenine (Guchhait, 1976). Further evidence to support the notion of endogenous production of 5‐MeO‐DMT and its potential physiological function in humans is needed.

## 2 PHARMACOLOGY AND METABOLISM

Two receptor binding studies based on human cloned receptors mutually revealed that 5‐MeO‐DMT is primarily nonselective serotonin (5‐HT) receptor agonist (Halberstadt et al., 2012; Ray, 2010), while additional binding to the 5‐HT‐transporter and dopamine receptors (Ray, 2010) or the noradrenergic transporter (Halberstadt et al., 2012) may contribute to its action. Both studies converged on the finding that 5‐MeO‐DMT has the highest binding affinity for the 5‐HT1A receptor (i.e., 1.9–3 nM) and a 300–1000 fold higher selectivity compared with the 5‐HT2A receptor (Halberstadt et al., 2012; Ray, 2010). This is noteworthy, as for most psychedelics, the functional and experiential effects in humans appear to be mediated primarily via activation of the serotonergic 5‐HT2A receptor (Barker, 2018; Madsen et al., 2019; Nichols, 2016; Vollenweider et al., 1998). 5‐HT1A receptors have been implicated in mood regulation (DeLorenzo et al., 2016) and the control of the autonomic nervous system (Youn et al., 2013). Specific stimulation of 5‐HT1A receptors produces sympathoinhibition and decreases blood pressure and heart rate, whereas ligation of 5‐HT2A receptors produce sympathomimetic effects such as increased heart rate, vasomotor tone, and blood pressure (Kaumann & Levy, 2006; Ramage, 1990). Preclinical studies showed that some 5‐MeO‐DMT induced behaviors such as decreased locomotor activity, investigatory behavior, and disturbed thalamocortical oscillations are selective to 5‐HT1A receptor activation as these can be attenuated by a selective 5‐HT1A antagonist but not by the 5‐HT2A antagonist (Krebs‐Thomson et al., 2006; Riga et al., 2016; Riga et al., 2018). Other autonomous effects of 5‐MeO‐DMT such as hyperthermia can be attenuated both by 5‐HT1A and 5‐HT2A receptor antagonists (Jiang et al., 2015). 5‐MeO‐DMT induces a signature head to twitch response in animals that is common to all psychedelics and that can only be blocked with 5‐HT2A antagonists (Halberstadt, 2016; Halberstadt et al., 2011). Other behavioral effects of 5‐MeO‐DMT in preclinical studies, including locomotor activity and exploration, sensorimotor gating, and those associated with 5HT behavioral syndrome, appear to be primarily modulated by 5‐HT1A, although 5‐HT2A activation is involved (Berendsen et al., 1989; Eison & Wright, 1992; Ermakova et al., 2021; Halberstadt & Geyer, 2011; Krebs‐Thomson et al., 2006; Lucki et al., 1984; Smith & Peroutka, 1986; Tricklebank et al., 1985).

**FIGURE 2:**
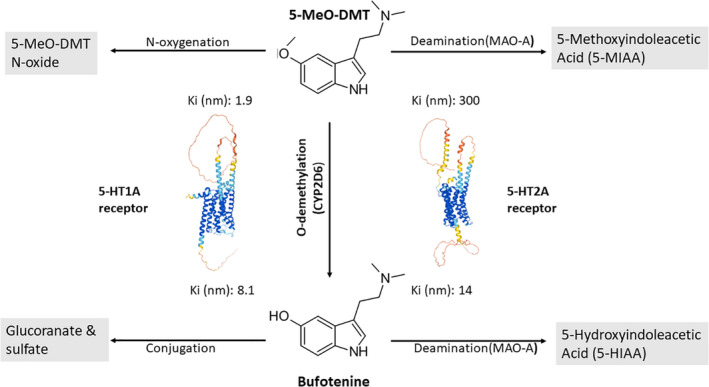
Biotransformation of 5‐MeO‐DMT and its metabolite bufotenine and their mean binding affinities (Ki) for 5‐HT1A (Hamon et al., 1986; Halberstadt et al., 2012) and 5‐HT2A receptors (Halberstadt et al., 2012; McKenna & Peroutka, 1989)

5‐MeO‐DMT is primarily inactivated through a deamination pathway mediated by monoamine oxidase A (MAO‐A), and it is O‐demethylated by cytochrome P450 2D6 (CYP2D6) enzyme to produce an active metabolite, bufotenine (Yu et al., 2003; Yu et al., 2004). The latter binds with a higher affinity to the 5‐HT2A receptor than 5‐MeO‐DMT (Glennon et al., 1994; McBride, 2000; McKenna & Peroutka, 1989; Spencer et al., 1987) and also displays a high affinity for the 5‐HT1A receptor (Hamon et al., 1986). Concurrent use of 5‐MeO‐DMT with drugs or plants containing an MAO inhibitor (MAOI) can block its biotransformation and increase exposure to 5‐MeO‐DMT leading to enhanced and prolonged behavioral effects or even hyperserotonergic effects (Shen et al., 2010). A summary of the biotransformation of 5‐MeO‐DMT and bufotenine and their affinity for 5‐HT1A and 5‐HT2A receptors is given in Figure 2.

## 3 SUBJECTIVE EFFECTS, ROUTE OF ADMINISTRATION, DURATION, AND MAGNITUDE

Effects following administration of 5‐MeO‐DMT are similar to those of other tryptamine psychedelics such as psilocybin and DMT and are known to include a diverse set of acute subjective effects including visual, auditory, and time perception distortions, emotional experiences, and memory impairment (Barsuglia et al., 2018; Nichols, 2016; Ott, 2001; Shulgin & Shulgin, 1997b). 5‐MeO‐DMT is known to induce intense mystical‐type or “peak” experiences as well as feelings of ego dissolution (Davis et al., 2018; Metzner, 2013; Ott, 2001; Shulgin & Shulgin, 1997a; Uthaug et al., 2018; Uthaug et al., 2019; Uthaug, Lancelotta, Szabo, et al., 2020). Components of a mystical experience typically involve an authoritative sense of unity or connectedness accompanied by feelings of reverence, positively valenced feelings such as love or peace, alterations to the sense of both time and space, and difficulty with putting the experience into words (Barrett et al., 2015; Davis et al., 2019). Reports of ego dissolution are often described as a sense of oneness with the universe or the experience of relaxed boundaries between the self and the world (Uthaug et al., 2018; Uthaug et al., 2019; Uthaug, Lancelotta, Szabo, et al., 2020). The 5‐MeO‐DMT experience contrasts with the DMT experience, as the latter is known to produce particularly vivid and complex visual imagery rather than marked ego dissolution (Barker, 2018). Inhalation of smoked or vaporized secretion from the *Incilius alvarius* toad (50 mg of bufotoxin, estimated 5‐MeO‐DMT content 5–7 mg) has been found to occasion mystical experiences of similar in intensity to high‐dose psilocybin, another psychedelic tryptamine popularly found in “magic mushrooms,” but with a much shorter duration of action (Barsuglia et al., 2018).

Acute adverse effects include fear, sadness, anxiety, confusion, fatigue, crying, paranoia, trembling, vomiting, nausea, headache, pressure on the chest or abdomen and loss of body perception (Barsuglia et al., 2018; Davis et al., 2018; Davis et al., 2019; Reckweg et al., 2021; Uthaug, Lancelotta, Ortiz Bernal, et al., 2020; Uthaug, Lancelotta, Szabo, et al., 2020) Subacute effects include flashbacks, *i.e*. short re‐experiencing of some of the subjective 5‐MeO‐DMT effects and reactivations, *i.e*. brief (in order of seconds) sensory disturbances such as flashes of light (Reckweg et al., 2021; Uthaug, Lancelotta, Ortiz Bernal, et al., 2020). Reactivations have been reported during the week after 5‐MeO‐DMT exposure (Reckweg et al., 2021; Uthaug, Lancelotta, Ortiz Bernal, et al., 2020; Uthaug, Lancelotta, Szabo, et al., 2020), and may occur more frequently after vaporization as compared to intramuscular administration (Uthaug, Lancelotta, Ortiz Bernal, et al., 2020). Of particular importance, the reactivation phenomenon has been reported primarily as a positive or neutral experience from large samples of recreational and ceremonial users (Davis, 2019). Rare cases of psychosis have also been reported (Metzner, 2013; Shulgin & Shulgin, 1997b), sometimes when used in combination with other tryptamines (Sauras Quetcuti et al., 2019).

5‐MeO‐DMT is orally inactive (Shulgin & Shulgin, 1997a) as it is rapidly metabolized by monoamine oxidase enzymes in the gut and liver (Shen et al., 2010). Therefore, 5‐MeO‐DMT is usually administered parenterally through smoking or inhalation of vapor or less commonly via intravenous, intramuscular, rectal, sublingual, or intranasal applications (Davis et al., 2018; Metzner, 2013; Weil & Davis, 1994). Smoking and vaporization are the most popular routes of administration because these are relatively easy and accessible and well documented in unofficial user guides (Most, 1984). Observational research has demonstrated that smoking/inhalation of 5‐MeO‐DMT vapor causes a very rapid onset of subjective effects, reaching peak effects in a matter of seconds (Uthaug, Lancelotta, Ortiz Bernal, et al., 2020) and lasting for 15–20 min (Weil & Davis, 1994). In observational studies, subjective effects induced by smoked 5‐MeO‐DMT are characterized by an intense psychedelic experience, with many participants reporting prominent ratings of ego dissolution and oceanic boundlessness (Barsuglia et al., 2018; Uthaug et al., 2019; Uthaug, Lancelotta, Szabo, et al., 2020), experiences characterized by disruption of self‐world boundaries, and feelings of unity with others and one’s surroundings. The magnitude of the experience however may vary considerably between individuals, as about 20–30% of participants in 5‐MeO‐DMT ceremonies reported a low to medium psychedelic experience (Uthaug et al., 2019; Uthaug, Lancelotta, Szabo, et al., 2020). This variability in psychedelic experience may have been caused by differences in doses administered at ceremonies, inhalation techniques, and the actual concentration of 5‐MeO‐DMT used by different facilitators.

Conversely, intramuscular injection (IM) has been reported to produce a slower onset of subjective effects, beginning between 1 and 6 min after injection, and lasting for up to 60 min (Uthaug, Lancelotta, Ortiz Bernal, et al., 2020). Subjective reports suggest that subjective experiences after IM administration are more gentle, more gradual, and less intense compared with vaporized 5‐MeO‐DMT and produces fewer reactivations (Uthaug, Lancelotta, Ortiz Bernal, et al., 2020), which are flash‐memories of the 5‐MeO‐DMT experiences that can occur infrequently during 1–2 weeks after an experience with 5‐MeO‐DMT (Davis & Lancelotta, 2018).

In his book *The Toad and The Jaguar*, Ralph Metzner summarized field and under‐ground reports on the subjective effects of 5‐MeO‐DMT when administered intranasally (Metzner, 2013). Intranasal administrations of 5‐MeO‐DMT purportedly induce a slower onset (i.e., 5–7 min) of subjective effects because of delayed absorption of the material through the mucus membrane. Dissociative effects of intranasal administrations are reportedly less pronounced but more prolonged (i.e., 45–60 min) compared with smoking or vaporization. Snuffing synthetic 5‐MeO‐DMT into the nasal passage was associated with mild and transient burning sensations while snuffing of toad secretion, was noted to be toxic and quite unpleasant (Metzner, 2013).

Conceptually, all formulations (smoked, vaped, IM, intranasal, intravenous) allow for high bioavailability of 5‐MeO‐DMT because they avoid first‐pass metabolism, with intravenous formulations providing maximal (100%) bioavailability. IM, intravenous, intranasal, and vaped administration offer easy control of dose delivery as compared to smoking. IM and intranasal administration of 5‐MeO‐DMT produce a relatively gentle experience with a slow onset and longer duration (Sherwood et al., 2019), while smoked and vaporized administration provides fast onset of subjective experiences with high intensity and short duration. At present, biopharmaceutical companies with an interest in 5‐MeO‐DMT are exploring and developing vaporized, intranasal, IM, and intravenous formulations for delivering 5‐MeO‐DMT (see Table 1).

**TABLE 1:** Summary of completed and ongoing clinical trials with 5‐MeO‐DMT formulations and formulations under development

Study	5‐MeO‐DMT formulation	Sample	Aim	Sponsor	Study site	Clinical trial	Status	Ref
Phase 1	Inhalable	Healthy volunteers (*N* = 22)	Safety	GH research	Maastricht University, The Netherlands	NCT04640831	Completed	(Reckweg et al., 2021)
Phase1/2a	Inhalable	Patients with TRD (*N* = 16)	Safety/ efficacy	GH research	Maastricht University, The Netherlands	NCT04698603	Completed	–
Phase 1	Inhalable	Healthy volunteers (*N* = 46)	Safety	GH Research	–	–	Completed	GH research
Phase 1	Intranasal	Healthy volunteers (*N* = 42)	Safety	Beckley Psytech	King's College London, UK	NCT05032833	Ongoing	–
–	IV/intranasal	–	–	GH research	–	–	–	GH research
–	Termosensitive nasal gel	–	–	Biomind	–	–	–	Biomind
–	Compositions containing purified toad secretion tryptamines	–	–	CaaMTech		–	–	CaaMTech
–	Oral (analog 5‐MeO‐DPT)	–	–	CaaMTech		–	–	CaamTech
–	Not specified	–	–	Alvarius Pharmaceuticals	–	–	–	Alvarius
–	Yeast strains			CB Therapeutics				CB therapeutics
–	API 5‐MeO‐DMT succinate	–	–	Usona Institute		–	–	(Sherwood et al., 2020; Sherwood et al., 2019)

## 4 MOTIVATION OF USE AND MENTAL HEALTH OUTCOME

Patterns of use, motivations for consumption, and subjective experiences associated with 5‐MeO‐DMT use have been examined using a web‐based retrospective survey (N = 515) by Davis and colleagues (Davis et al., 2018). Findings revealed that respondents consumed 5‐MeO‐DMT infrequently, less than once a year, and less than four times in a lifetime, mainly for spiritual exploration. The majority (90%) reported moderate‐to‐strong mystical‐type experiences (ineffability, timelessness, awe/amazement, experience of pure being/awareness), and relatively fewer (37%) experienced challenging experiences. Less than half (39 %) reported repeated consumption during the same session, and very few reported drug craving/desire (8%), legal (1%), medical (1%), or psychiatric (1%) problems related to use.

Prospective observational studies on the naturalistic use of synthetic 5‐MeO‐DMT and toad secretion containing 5‐MeO‐DMT in healthy volunteers have demonstrated immediate and lasting improvements in self‐reported ratings of depression, anxiety, stress, mindfulness‐related capacities, and satisfaction with life, after a single inhalation of the substance (Uthaug et al., 2019; Uthaug, Lancelotta, Szabo, et al., 2020). The first prospective study with 5‐MeO‐DMT (Uthaug et al., 2019) demonstrated that, compared with baseline, participants (*n* = 42) reported reductions of depression, anxiety, and stress, as well as increases in mindfulness‐related capacities and satisfaction with life, 24‐h postintake of toad venom containing 5‐MeO‐DMT. Such improvements in mood, mindfulness‐related capacities, and life satisfaction were found to persist up to 4 weeks after intake. It was further found that participants who experienced higher levels of ego dissolution reported higher levels of satisfaction with life and lower levels of depression and stress 24 h after the drug experience. A follow‐up study by the same group (Uthaug, Lancelotta, Szabo, et al., 2020) demonstrated that mindfulness‐related capacities were enhanced, and feelings of depression were reduced immediately after intake of synthetic 5‐MeO‐DMT compared with baseline (*N* = 11). Seven days postintake, mindfulness‐related capacities remained enhanced and feelings of anxiety and stress were significantly reduced compared with baseline (Uthaug, Lancelotta, Szabo, et al., 2020). Again, it was found that the more of an acute experience of ego dissolution, the more of a decrease in feelings of depression, anxiety, and stress, and the more of an increase in mindfulness‐related capacities, both directly after the session and at the 7‐day follow‐up (Uthaug, Lancelotta, Szabo, et al., 2020). Taken together, results from these two prospective studies demonstrate fast‐acting, and in some cases, immediate, improvements in mood in self‐reported healthy volunteers, who ingested the substance in a naturalistic environment. Importantly, these studies also suggest a relationship between the strength of the acute psychedelic experience and the magnitude of persisting mood changes.

Further observational studies have reported reductions in the symptomology of a range of different diagnosed psychiatric disorders after ingestion of 5‐MeO‐DMT. Davis et al. (2018) employed a cross‐sectional survey to characterize patterns of use and assess potential risks and benefits associated with 5‐MeO‐DMT use. Of the respondents that indicated to have been diagnosed with a psychiatric disorder, marked improvements were reported in the following conditions: PTSD (79%), depression (77%), anxiety (69%), substance use problems (63%), and obsessive‐compulsive disorder (53%), with a notably small proportion (2–10%) reporting a worsening of these conditions. In a separate cross‐sectional survey, the same group (Davis et al., 2019) demonstrated similar reports of improvements in depression and anxiety conditions amongst respondents following 5‐MeO‐DMT use in a group setting. Namely, the authors demonstrated that of those with depression (*n* = 149), 80% reported an improvement in their conditions following 5‐MeO‐DMT use, whereas 17% reported no change in depression, and 3% reported a worsening of their depression. In those who had anxiety (*n* = 173), 79% reported an improvement in their condition following 5‐MeO‐DMT use, whereas 19% reported no change in anxiety, and 2% reported a worsening of their anxiety. Similar to the observational studies in healthy participants, Davis and colleagues (Davis et al., 2019) found that the stronger the mystical experience, the higher the ratings of spiritual significance and personal meaning of the acute 5‐MeO‐DMT experience, the higher the reported improvements in depression and anxiety. The authors further noted that out of the entire sample, only 7 individuals reported using 5‐MeO‐DMT specifically to help with their depression or anxiety, highlighting that the reported improvements of most respondents were unintended.

## 5 NEUROPHYSIOLOGICAL EFFECTS

The full spectrum of the potential (neuro)physiological effects of 5‐MeO‐DMT in mammals is yet to be understood. Here, we will mainly focus on two therapeutically relevant physiological domains: the neuroendocrine and immunological effects of this indolealkylamine hallucinogen. We will first discuss the possible neuroendocrine effects of 5‐MeO‐DMT on mammalian physiology. Then we will focus on the consequences of its administration in inflammatory regulation and immune functions in preclinical and observational studies.

### 5.1 Neuroendocrine effects

Initial studies in the late 1970s and early 1980s focused on the effect of 5‐MeO‐DMT on luteinizing hormone (LH) and prolactin (PRL) released by the pituitary gland in rodent models (Kuhn et al., 1981; Lenahan et al., 1986; Seeman & Brown, 1985; Simonovic & Meltzer, 1979; Simonovic & Meltzer, 1983). These early studies mapped the in vivo effects of 5‐MeO‐DMT administration on the level of neuroendocrine factors involved in the regulation of hormonal cycles, metabolism, and systemic immune functions. While 5‐MeO‐DMT did not affect LH and estrogen levels (Lenahan et al., 1986), it strongly promoted the release of PRL in rats (Kuhn et al., 1981; Simonovic & Meltzer, 1979). The onset of this stimulatory effect on PRL response was slow and gradual, possibly because of the sensitization of pituitary‐related serotonergic mechanisms. Later, a biphasic effect of 5‐MeO‐DMT on PRL secretion was described that involved the originally observed, initial, secretion‐stimulating effect followed by an inhibitory effect on PRL release in the long run (Simonovic & Meltzer, 1983; Seeman & Brown, 1985). The initial pro‐secretory effects of 5‐MeO‐DMT were hypothesized to be as a result of its ability to activate postsynaptic 5‐HT receptors. On the other hand, the subsequent inhibitory effect on PRL secretion was found to be based on increased functional activity of tuberoinfundibular dopamine neurons (Simonovic & Meltzer, 1983). Furthermore, Seeman and Brown (Seeman & Brown, 1985) also compared the neurohormonal effects of 5‐MeO‐DMT with two other close tryptamine analogs, bufotenin, and DMT. They found that the most potent pro‐secretory effects on PRL levels were observed in the case of 5‐MeO‐DMT administration, followed by bufotenin, and finally by DMT. This latter phenomenon was, at least partly, because of the different in vivo stability of these tryptamines, as well as individual characteristics related to their intraparenchymal transport via the blood‐brain barrier. Another important message of these early animal studies was that the observed, 5‐MeO‐DMT‐mediated neuroendocrine response was centrally mediated, and did not involve activation of peripheral 5‐HT receptors.

An important aspect of the possible therapeutic use of serotonergic tryptamines is their modulatory effect on neuroplasticity. Neuropsychiatric diseases, such as major depressive disorder (MDD), anxiety, PTSD, or addiction have high comorbidity (Kelly & Daley, 2013) and share common underlying neural principles (Arnsten, 2009; Russo et al., 2009; Russo & Nestler, 2013). A plethora of previous reports suggests that decreased neuroplasticity and neural atrophy in the prefrontal cortex (PFC) play an essential role in the general pathophysiology of these disorders (Duman & Aghajanian, 2012; Duman et al., 2016; Izquierdo et al., 2006; Pittenger & Duman, 2008; Russo & Nestler, 2013; Qiao et al., 2016). These disruptive structural changes in the PFC could be rectified by compounds that can promote structural and functional neural plasticity (Castren & Antila, 2017; Cramer et al., 2011; Kolb & Muhammad, 2014; Duman, 2002; Krystal et al., 2009). In essence, such compounds would provide a general solution to treating all these related disorders (Ly et al., 2018). Serotonergic psychedelics, entactogens, and ketamine have been shown to mediate rapid onset, long‐lasting anxiolytic, and antidepressant effects in humans following the administration of a single dose (Bouso et al., 2008; Carhart‐Harris & Goodwin, 2017; Grob et al., 2011; Mithoefer et al., 2013; Mithoefer et al., 2016; Sanches et al., 2016; Osorio Fde et al., 2015), or multiple therapeutic doses in randomized controlled trials (Davis et al., 2021; Carhart‐Harris et al., 2021). This evident therapeutic effect was also reported in treatment‐resistant populations of patients (Mithoefer et al., 2011; Oehen et al., 2013; Carhart‐Harris, Bolstridge, et al., 2016; Carhart‐Harris et al., 2017; Rucker et al., 2016). The neuroplasticity‐promoting effect of several serotonergic psychedelics in the PFC has recently been reported by Ly et al., including those that are closely related to 5‐MeO‐DMT (Ly et al., 2018). Furthermore, a recent study, using *in vitro*‐differentiated human embryonic stem cell‐derived cerebral organoids, found similar modulatory effects of 5‐MeO‐DMT on molecular pathways involved in neuroplasticity (Dakic et al., 2017). In addition, another group reported in vivo pro‐neuroplastic effects of a single dose of intracranially administered 5‐MeO‐DMT in mice (Lima da Cruz et al., 2018).

One of the key neuroplasticity‐modulating neuroendocrine factors is the Brain‐derived neurotrophic factor (BDNF). BDNF is a crucial neurotrophin that is involved in both the maintenance of brain homeostasis and in multiple neuropathologies (Lu et al., 2013; Leal et al., 2014; Kowianski et al., 2018). Multiple evidence suggests that psychedelics and related compounds could prove useful in the effective modulation of BDNF/neurotrophin levels, and thus in the alleviation of the physiological, behavioral, and cognitive symptoms of several neurodegenerative disorders (Saeger & Olson, 2021). A single, low dose of LSD was shown to acutely increase BDNF levels in healthy volunteers (Hutten et al., 2021). Likewise, a double‐blind randomized placebo‐controlled ayahuasca clinical trial reported the modulation of BDNF in patients with MDD. The authors reported significant modulation of serum BDNF by a single dose of ayahuasca, which suggests a link between the previously observed antidepressant effects of the psychedelic brew (de Almeida et al., 2019). Since the primary psychoactive component of ayahuasca is DMT, a close structural analog of 5‐MeO‐DMT, it is tempting to speculate that 5‐MeO‐DMT also has potential systemic BDNF‐modulatory and neuroplasticity‐promoting effects in humans.

### 5.2 Effects on immune regulation and inflammation

To understand the potential effects of 5‐MeO‐DMT on immune homeostasis, we need to consider two major, down‐stream effector mechanisms that may alter the inflammatory/immune status of the organism as a consequence of indolealkylamine administration. These two proposed major mechanisms are i) the influence of 5‐MeO‐DMT on systemic neuroendocrine regulation and ii) its modulatory effect on immune cells and on inflammatory and immune‐related intracellular pathways via 5‐HT2A and Sig1R. In the following, we will discuss these two effector mechanisms in detail by reviewing the available literature to date.

**FIGURE 3:**
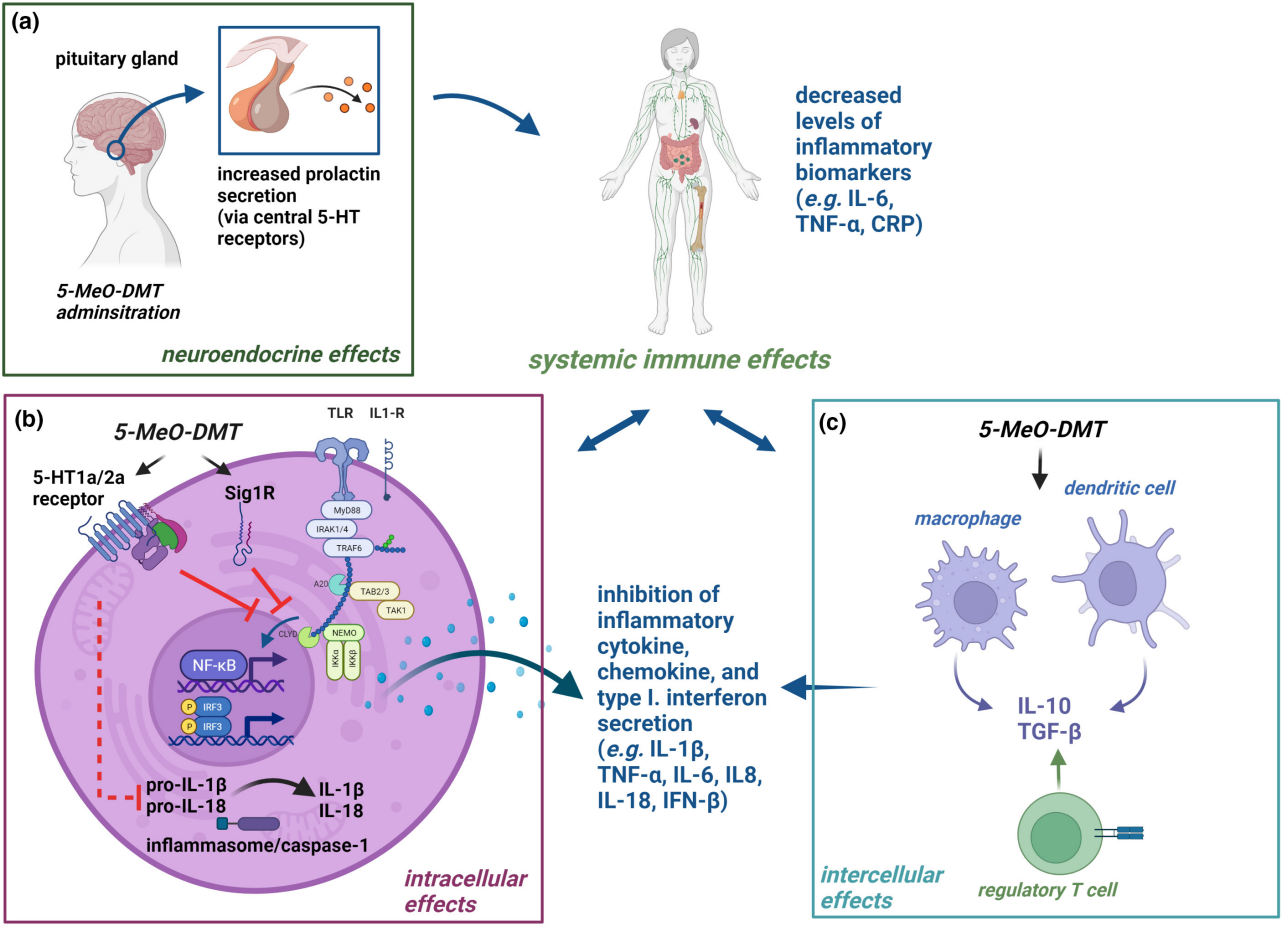
The putative physiological effects of 5‐MeO‐DMT administration on the neuroendocrine and immune systems. (a) The systemic physiological effects of 5‐MeO‐DMT may include direct modulation of prolactin secretion via central hypothalamic serotonin receptors. This can result in systemic anti‐inflammatory and immunomodulatory effects through prolactin receptor‐expressing immune cell types. (b) Serotonin 1A and 2A (5‐HT1a/2a), Toll‐like (TLR), and inflammatory cytokine receptors (e.g., IL‐1β receptor, IL1‐R) are expressed on the cell surface, while sigma‐1 receptors (Sig1R) are localized intracellularly mainly at the mitochondrion‐associated endoplasmic reticulum interface. TLRs and other pattern recognition receptors recognize various sets of pathogenic structures and transduce signals through the NF‐κB/IRF pathways (e.g., IRF3). IL1‐R is specifically modulated by inflammatory cytokines of the IL‐1 family (e.g., IL‐1β). The activation of TLRs/IL1‐Rs results in down‐stream signaling through the MyD88 adaptor proteins. This receptor–adaptor interaction leads to the activation of the essential coadaptors IRAK1/4 and TRAF6 and leads to the subsequent phosphorylation of several effector pathways/regulators, such as IRF3 or NF‐κB (via NEMO/IKKα/IKKβ). These transcription factors then translocate to the nucleus regulating the expression of type I IFN, chemokine, and inflammatory cytokine genes, such as IFNβ, IL‐1β, IL‐6, IL‐8, IL‐18, and TNF‐α. 5‐MeO‐DMT may interfere with these pathways via down‐stream signals from 5‐HT1a, 5‐HT2a, and Sig1R. The activation and assembly of the inflammasome complex by PAMPs/DAMPs lead to the recruitment and activation of caspase‐1, and the subsequent cleavage of pro‐IL‐1β and IL‐18 to the biologically active, released form (IL‐1β and IL‐18). 5‐MeO‐DMT may also inhibit this process through an unknown mechanism(s). (c) 5‐MeO‐DMT can also directly modulate the production of anti‐inflammatory cytokines, such as IL‐10 and TGF‐β, by 5‐HT1a/2a and/or Sig1R expressing immune cell types thereby inhibiting local and systemic inflammatory processes. Abbreviations: 5‐HT: serotonin; IL: interleukin; TNF: tumor necrosis factor; CRP: C‐reactive protein; MyD88: Myeloid differentiation primary response 88 adaptor protein; IRAK: Interleukin‐1 receptor‐associated kinase; TNF receptor‐associated factor 6 adaptor protein; IRF: Interferon regulatory factor; NF‐κB: Nuclear factor kappa‐light‐chain‐enhancer of activated B cells transcription factor; NEMO: NF‐kappa‐B essential modulator; IKKs: inhibitory‐κB kinases; IFN: interferon; TGF‐β: Transforming growth factor‐beta; Red T‐arrows represent inhibitory effects. Created with BioRender.com

As discussed above, 5‐MeO‐DMT has a formidable modulatory capacity in promoting PRL release via central 5‐HT receptors. This effect appears to be biphasic in rodent models with increased serum PRL levels in the early acute phase and gradually decreased levels in the long run. PRL is involved in multiple physiological processes including the regulation of metabolism, cellular growth, and apoptosis, as well as immune functions (Bole‐Feysot et al., 1998; Freeman et al., 2000). Initially, PRL was considered to be an immunostimulatory factor with potentially deleterious effects in autoinflammatory and autoimmune pathologies, as hyperprolactinemia was a signature biomarker in different autoimmune diseases (Borba et al., 2019). In the last decade, however, this common view has been challenged by findings demonstrating that PRL has no effect on the symptom development and severity of experimental autoimmune encephalomyelitis, a standard animal model for multiple sclerosis, and can even be protective in rheumatoid arthritis (Costanza et al., 2015). Moreover, recent findings have shown that PRL has a direct inhibitory effect on key factors and pathways involved in inflammation, such as nuclear factor kappa‐B (NF‐κB) signaling, and on the secretion of inflammatory cytokines, for example, interleukin‐1β (IL‐1β), IL‐6, tumor necrosis factor‐alpha (TNF‐α) (Olmos‐Ortiz et al., 2019). Furthermore, it also exerts anti‐inflammatory effects in the central nervous system by regulating microglial functions and has been described as a neuroprotective and neuroplasticity‐promoting factor (Ramos‐Martinez et al., 2021). Since 5‐MeO‐DMT strongly modulates the circulating levels of PRL, it may have anti‐inflammatory and immune regulatory properties at both systemic and intracellular levels, for example, via the indirect inhibition of NF‐κB signaling and pro‐inflammatory cytokine production (Figure 3). Because of its biphasic effect on PRL secretion in vivo, the immune‐modulatory potential of 5‐MeO‐DMT is expected to be stronger in early acute scenarios (0–4 h after administration) and is gradually down‐regulated in the long run (>12 h). Nonetheless, in the lack of clinical data, it would be unwise to extrapolate these preclinical findings to human physiology. Importantly, however, in a recent observational study, we documented significant decreases in IL‐6 and increases in cortisol levels in the saliva of healthy volunteers following acute 5‐MeO‐DMT administration (Uthaug, Lancelotta, Szabo, et al., 2020). As the secretion of PRL and cortisol are tightly coregulated, and both hormones exhibit potent immunomodulatory properties (Henry, 1992; Levine & Muneyyirci‐Delale, 2018), these data may support the validity of the PRL‐effector hypothesis in humans. However, in the absence of controlled clinical trials using larger patient samples, the current findings regarding the acute and systemic anti‐inflammatory effects of 5‐MeO‐DMT have to be interpreted with caution.

The specific effects of 5‐MeO‐DMT on human immune cells and on intracellular inflammatory pathways have been explored in preclinical studies. Inflammation is an immediate response to invading microbes or tissue damage mediated by the innate immune system, an ancient host defense mechanism. The detection of potentially dangerous, nonself, pathogen‐associated molecular patterns (PAMPs) is done by pattern recognition receptors (PRRs) that are widely expressed in immune cells and in various tissues. PAMPs are evolutionarily conserved patterns commonly found in larger microbial taxa. Recognition of these molecular motifs by PRRs typically initiates NF‐κB‐mediated inflammatory cytokine, chemokine, or interferon responses that are specified by the type of microbe. In the recent two decades, a number of various PRRs have been identified, such as toll‐like and C‐type lectin receptors (TLRs and CLRs), cytosolic nucleotide‐binding oligomerization domain‐containing (NOD)‐like receptors (NLRs), and many others (Thaiss et al., 2016; Riera Romo et al., 2016). Specific activation of these PRRs by their microbial ligands activate the NF‐κB and the interferon (IFN)‐regulatory factor 3/7 (IRF3/7) pathways, and the mitogen‐activated protein kinases (MAPKs), for example, p38, ERK1/2, c‐Jun N‐terminal kinase (JNK) (Kawai & Akira, 2008; Benko et al., 2008; Szabo & Rajnavolgyi, 2013). This process leads to the expression of a common set of genes that define the secretion of inflammatory cytokines, chemokines, and co‐stimulatory molecules, which are critical for the orchestration of both innate and adaptive immune responses including but not limited to IL‐1β, IL‐6, and TNF‐α (Figure 3). The modulation of the pathways above is essential in antimicrobial immune responses but can also be involved in autoimmune processes where aberrant inflammation causes chronic and severe damage to self‐tissues (Doria et al., 2012).

Some of the PRRs (e.g., the NLR family) are involved in the constitution and activation of the inflammasome, an important innate immune effector mechanism. Inflammasomes are large and complex, multicomponent platforms that regulate caspase‐1 activation. Caspase‐1 is a proteolytic enzyme that controls the cleavage of pro‐inflammatory IL‐1 family cytokines, such as IL‐1β and IL‐18 (Figure 3), as well as modulates pyroptosis, an inflammatory form of cell death (Rathinam et al., 2012; Rathinam & Fitzgerald, 2016). Cytosolic detection of PAMPs triggers the rapid assembly of the inflammasome complex. In addition to this “canonical” form of inflammasome and caspase‐1 activation, self‐derived endogenous damage‐associated molecular patterns (DAMPs) can also lead to the activation of inflammasomes. DAMPs can be produced during tissue damage, metabolic dysregulation, and even by psychological stress (Iwata et al., 2016) and can drive pathological “sterile inflammation,” a phenomenon that is occurring in the absence of pathogenic microbes (Latz et al., 2013). Chronic inflammation provoked by either exogenous or endogenous stimuli can elicit substantial tissue damage that may lead to autoimmunity. Thus, innate immunity can play an important role in the etiology of various autoimmune diseases by initiating and sustaining autoinflammatory processes, decreasing the immune tolerance threshold, and contributing to the development of long‐term adaptive immune responses against self‐tissues, such as the brain parenchyma (Doria et al., 2012).

The available literature on the immunomodulatory properties of psychedelic tryptamines is scarce and mostly confined to preclinical studies (Flanagan & Nichols, 2018; Kyzar et al., 2017). A small number of existing studies, by combining *in vitro* assays and *in silico* analyses, have demonstrated the anti‐inflammatory properties of DMT and 5‐MeO‐DMT (Tourino et al., 2013; Szabo et al., 2014; Dakic et al., 2017). In some of these studies, 5‐MeO‐DMT was found to exert potent anti‐inflammatory activity through the Sig1R of human cerebral organoids (Dakic et al., 2017), and in human monocyte‐derived dendritic cells (moDCs), a cell type critically involved in both innate and adaptive immunity (Szabo et al., 2014). 5‐MeO‐DMT and DMT treatment of immune‐primed human primary moDCs led to significant inhibition of inflammatory cytokine and chemokine secretion including IL‐1β, IL‐6, TNF‐α, and IL‐8/CXCL8. On the other hand, both 5‐MeO‐DMT and DMT were able to upregulate specific mRNA transcripts and secretion of the anti‐inflammatory cytokine IL‐10 in the treated cells. The tryptamines also exhibited an effective blocking capacity at the level of adaptive immunity by inhibiting T helper cell 1 and 17 priming by 5‐MeO‐DMT and DMT‐treated moDCs. Furthermore, 5‐MeO‐DMT appeared to be slightly more potent than DMT with regard to its immunomodulatory potential, although the differences were only trending and not statistically significant. The observed anti‐inflammatory modulation was dominantly mediated by Sig1R, and partly by other receptors, most likely by 5‐HT2A (Szabo, 2015; Szabo et al., 2014). Furthermore, these findings are in good agreement with the results of our recent report on a small cohort of healthy volunteers, where a single dose of 5‐MeO‐DMT caused a rapid decrease in salivary IL‐6 levels (Uthaug, Lancelotta, Szabo, et al., 2020). In the same study, we did not observe any effect of 5‐MeO‐DMT on salivary IL‐1β and TNF‐α, most probably because of the study design that only allowed us to probe into early acute alterations. Since the biological half‐life of both IL‐1β and TNF‐α are relatively long, the sample collection between baseline and post‐administration (~1.5–2.5 h) did not make it possible to detect any changes that presume degradation/down‐regulation of these cytokines. Significant increases in salivary cortisol were also observed following 5‐MeO‐DMT exposure that may support both the PRL‐effector hypothesis as well as the intracellular immunomodulatory effects via Sig1R, as discussed above (Uthaug, Lancelotta, Szabo, et al., 2020; Szabo, 2015).

The anti‐inflammatory and immune effects of 5‐MeO‐DMT can therefore be mediated by i) systemic neuroendocrine feedback loops based on the activation of central 5‐HT receptors and the release of PRL by the pituitary, and cortisol by the adrenal glands. ii) 5‐MeO‐DMT can also exert direct inhibition on key inflammatory pathways, such as by blocking the signal transduction of the ‟master switch” transcription factor NF‐κB and associated pathways, *e.g*. MAPKs and IRFs, as suggested earlier (Kawai & Akira, 2008; Benko et al., 2008; Szabo & Rajnavolgyi, 2013). This latter may happen via competition for signaling adaptors and/or intracellular elements between Sig1R, 5‐HT2A, and PRRs. iii) The same competitive inhibition may also interfere with inflammasome activation, and thus the maturation and release of IL‐1β and IL‐18 (Figure 3). iv) Another alternative is the direct and specific upregulation and release of anti‐inflammatory cytokines, such as IL‐10, and possibly transforming growth factor‐beta (TGF‐β), which then subsequently down‐regulate inflammatory signals (Figure 3). v) Finally, epigenetic modifications as the down‐stream consequence of Sig1R and 5‐HT2A (and/or other serotonin receptor) activation may contribute to the long‐term immunomodulatory and pro‐neuroplastic effects of 5‐MeO‐DMT and probably of other tryptamines. This mechanism may underlie the observed, long‐term beneficial effects of a single dose of serotonergic tryptamine on the symptoms of depression and anxiety.

The systemic–physiological–neuroendocrine regulatory loops and the intracellular‐paracrine cytokine loops may also cross‐talk via 5‐HT2A, Sig1R, cytokine, and hormone receptors and their associated pathways following 5‐MeO‐DMT administration to dampen inflammatory responses, especially in the early acute phase (Figure 3). To date, only a handful of preclinical studies are available on the neuroendocrine and immunomodulatory capacity of 5‐MeO‐DMT. The biochemical and immunopharmacological versatility and documented anti‐inflammatory potential of this evolutionarily ancient tryptamine warrant further investigations. Human clinical trials testing the neuroendocrine, pro‐neuroplastic, and anti‐inflammatory effects of 5‐MeO‐DMT would be of great importance and would have numerous ramifications in neuropsychiatric disorders, and in future drug design.

## 6 PSYCHOLOGY VERSUS NEUROPHYSIOLOGY?

Potential mechanisms underlying positive mental health changes induced by psychedelics have been attributed to both the psychological psychedelic experience (Yaden & Griffiths, 2021) as well as the underlying neurophysiological mechanisms (Olson, 2021). In the psychological perspective, the experience of certain subjective psychedelic effects is deemed necessary to evoke a therapeutic response, whereas, in the neurophysiological perspective, the subjective experiences elicited by psychedelic substances are merely epiphenomena of the underlying neurobiological mechanisms, the latter which are conveying the beneficial effects of psychedelics. Both perspectives, however, are not necessarily mutually exclusive when explaining the long‐term beneficial effects of psychedelics including 5‐MeO‐DMT.

Core to the psychological perspective is the notion that a psychedelic‐occasioned mystical state of consciousness, or peak psychedelic experience, is the primary factor that mediates enduring positive effects in cognition, affect, behavior, and spirituality (Griffiths et al., 2006; Gasser et al., 2015; Roseman et al., 2018). This notion is also shared by patients treated with psychedelics, who acknowledge the importance of acute subjective effects in therapeutic outcomes as documented in qualitative interviews (Noorani et al., 2018; Belser et al., 2017). A recent review of 20 studies assessing the clinical response to psychedelics in patients with an addictive disorder, treatment‐resistant depression, and obsessive‐compulsive disorder provided further support and concluded that the main predictive factor of a response to a psychedelic is the intensity of the acute psychedelic experience (Romeo et al., 2021). This conclusion also seems in line with results from the 2 observational studies on 5‐MeO‐DMT (Uthaug, Lancelotta, Szabo, et al., 2020; Uthaug et al., 2019) that were reported above, in which higher intensities of the 5‐MeO‐DMT experience, namely higher ratings of the experience of ego dissolution, were associated with stronger long‐term improvements in mental health outcomes. However, the mechanism triggered by a mystical or peak experience leading to a change in down‐stream behavior and affect is still unclear. The mystical state experienced during psychedelics may facilitate psychological shifts such as enhancement of openness and increased psychological flexibility when coping with day‐to‐day stressors (Carhart‐Harris & Nutt, 2017). It is noteworthy in that respect that 5‐MeO‐DMT consistently changed the psychological state of participants in observational studies, as indicated by increases in nonjudgmental feelings about themselves and others, and reductions in overall feelings of stress and anxiety (Uthaug, Lancelotta, Szabo, et al., 2020; Uthaug et al., 2019). However, it cannot be excluded that lasting changes in the psychological state caused by 5‐MeO‐DMT also coincide with neurobiological changes that occur in parallel through 5‐HT1A and 5‐HT2A agonism. The latter has been associated with acute disintegration of normally highly organized activity within resting‐state networks (RSN), a simultaneous widening of dynamic repertoires of connectivity states, and increased coupling of RSNs that are usually anticorrelated (Palhano‐Fontes et al., 2015; Muller et al., 2018; Vollenweider & Preller, 2020; Mason, Kuypers, et al., 2020; Carhart‐Harris, Muthukumaraswamy, et al., 2016; Tagliazucchi et al., 2016). Likewise, anti‐inflammatory and immune effects as observed with 5‐MeO‐DMT (Uthaug, Lancelotta, Szabo, et al., 2020) and other psychedelics (Nichols, 2016) or increased expression of neurotrophic factors may contribute to the therapeutic effect of psychedelics as well (Romeo et al., 2021).

The neurophysiological perspective attaches more value to the quality of psychedelics to promote structural and functional neural plasticity in the brain through 5‐HT2A receptor‐mediated mechanisms (Ly et al., 2018) and considers psychedelic induced mystical experiences as a biomarker of 5‐HT2A receptor stimulation (Olson, 2021). It has been postulated that such neurobiological, or “psychoplastogenic”, effects can be decoupled from the subjective effects of psychedelics through chemical design without losing therapeutic potential (Olson, 2021). It is interesting in the present context that an engineered prototype of such a non‐hallucinogenic psychedelic is an analog of 5‐MeO‐DMT, termed tabernanthalog (Cameron et al., 2021; Olson, 2021). Tabernanthalog promoted structural neural plasticity, reduced alcohol‐ and heroin‐seeking behavior, and produced antidepressant‐like effects in rodents, suggesting that it might be effective at treating a range of neuropsychiatric diseases and addiction (Cameron et al., 2021). Future comparisons of the therapeutic effects of 5‐MeO‐DMT and tabernathalog in clinical studies will therefore be of particular interest when evaluating the contributory roles of subjective and neurobiological effects of 5‐MeO‐DMT, and psychedelics in general, to clinical response.

The moderating role of extra‐pharmacological factors such as set and setting for the experiential response to a psychedelic is also widely acknowledged in psychedelic research (Eisner, 1997; Hartogsohn, 2016; Mason, Dolder, & Kuypers, 2020; Uthaug et al., 2021). Set includes personal beliefs, attitudes, and motivations in relation to psychedelics, whereas setting refers to the external environment and context in which psychedelics are taken. Positive expectations, openness to the psychedelic experience and a trusted, supportive environment are key predictors of an optimal experience, whereas preoccupation with concerns, rigidity, lack of trust, and support increase the chances of an adverse experience (MacLean et al., 2011; Schenberg, 2018; Russ et al., 2019). This may be particularly true for experiences with 5‐MeO‐DMT that can be very intense and challenging (Uthaug, Lancelotta, Ortiz Bernal, et al., 2020). To examine this hypothesis, Sepeda and colleagues (Sepeda et al., 2020) used secondary data from a large survey study (Davis et al., 2018) to explore the acute and enduring effects of inhaled synthetic 5‐MeO‐DMT between individuals who used 5‐MeO‐DMT in a nonstructured context (NSC; *n* = 216) and a group of people who used it in a structured context (SC; *n* = 362). Both groups reported high ratings of mystical experiences; however, those who were administered 5‐MeO‐DMT in an SC reported significantly higher mystical experiences compared to those who were administered 5‐MeO‐DMT in an NSC. Additionally, the proportion of respondents who had a complete mystical experience was significantly lower in the NSC group (54%) compared to those in the SC group (83%). Ratings of the meaningfulness, spiritual significance, and well‐being associated with 5‐MeO‐DMT consumption were also significantly higher, and the intensity of challenging experiences was significantly lower, for those who were administered 5‐MeO‐DMT in an SC compared with those who were administered 5‐MeO‐DMT in an NSC.

Although these data regarding the supportive setting is associated with positive effects of 5‐MeO‐DMT consumption come from self‐report data, they help establish important theoretical support that can be tested in controlled studies with 5‐MeO‐DMT in well‐controlled environments in which the mental state of participants can be optimized in anticipation of 5‐MeO‐DMT treatment. Other published data (Lancelotta & Davis, 2020) from this group supported the notion that behavioral, psychological, and environmental strategies aimed at supporting the safety of the psychedelic experience can also be an important component of a 5‐MeO‐DMT experience. For example, findings suggest that employing such strategies is common among 5‐MeO‐DMT users and that ratings of the mystical experience were significantly higher among those who reported focusing on an intention, utilizing ceremonial or shamanic techniques, eliminating distractions, and meditating prior to the session. Findings also revealed that challenging experiences were rated significantly lower among those who reported preparing music for their session. Taken together, preparatory acts may include realistic projections of the actual experience with 5‐MeO‐DMT to increase realistic expectations and readiness, as well as psychological support and guidance by trained facilitators prior to, during, and after the 5‐MeO‐DMT experience, and providing psychological, emotional, and environmental safety for someone who will be administered 5‐MeO‐DMT is of critical importance.

## 7 THERAPEUTIC INDICATIONS OF 5‐MEO‐DMT AND CLINICAL DEVELOPMENT

A growing body of research is demonstrating the efficacy of psychedelic drugs such as psilocybin, ayahuasca, and lysergic acid diethylamide (LSD) in treating a variety of different psychiatric disorders, including treatment‐resistant depression, PTSD, substance use disorder, and anxiety‐related disorders (Vollenweider & Preller, 2020; Psiuk et al., 2021). However, systematic evidence of the therapeutic utility of 5‐MeO‐DMT is currently limited to anecdotal reports and observational studies in self‐selected healthy and clinical populations, who are using the drug in a natural environment (Lancelotta & Davis, 2020). As such, the current therapeutic potential of 5‐MeO‐DMT is mainly hypothetical (Ermakova et al., 2021) and based on preliminary evidence. As reviewed above, current therapeutic evidence stems from a small number of prospective observational studies and cross‐sectional surveys on the naturalistic use of synthetic 5‐MeO‐DMT and toad secretion containing 5‐MeO‐DMT in self‐selected samples. Findings of studies in self‐described healthy volunteers suggest immediate and lasting improvements in self‐reported feelings of depression, anxiety, stress, and satisfaction with life, after a single inhalation of the substance (Uthaug, Lancelotta, Szabo, et al., 2020; Uthaug et al., 2019). Additionally, reductions in the symptomatology of diagnosed depression and anxiety (Davis et al., 2018; Davis et al., 2019), as well as in a range of other psychiatric disorders such as PTSD, substance use problems, and obsessive‐compulsive disorder (Davis et al., 2018) after ingestion of 5‐MeO‐DMT have been reported. Although limited, the studies offer converging evidence of the potential ability of 5‐MeO‐DMT to provide fast‐acting, and potentially immediate, therapeutic relief for depression, anxiety, and stress‐related disorders (such as PTSD) in particular.

Improvements in mood in healthy volunteers and alleviation of psychiatric symptom severity in certain clinical populations after ingestion of 5‐MeO‐DMT are similar to results of observational and clinical studies assessing the therapeutic potential of the classic psychedelics psilocybin, LSD, and ayahuasca. As reviewed elsewhere (Vollenweider & Preller, 2020; Psiuk et al., 2021; Romeo et al., 2021) there is now a wealth of research demonstrating the safety of classic psychedelics, and a growing body of clinical work demonstrating their immediate and long‐lasting therapeutic efficacy. Thus, because of the apparent similar therapeutic profile as well as the extensive research behind the classic psychedelics, one may ask why investigations into 5‐MeO‐DMT are of interest. Here the duration of action of 5‐MeO‐DMT and the profile intensity of the 5‐MeO‐DMT experience may make it a particularly cost‐effective therapeutic agent.

After oral ingestion, the psychoactive effects of the classic psychedelics vary substantially: the full psychedelic effects of ayahuasca can last up to 4 h (Riba et al., 2003; Dos Santos et al., 2012), whereas psilocybin lasts approximately 6 h (Carbonaro et al., 2018), and LSD lasts approximately 12 h (Holze et al., 2021). Such long duration of action results in a day‐long therapy session, which includes clinical and infrastructural resources (Lancelotta & Davis, 2021). As such, it is likely that psychedelic‐assisted psychotherapy with these substances will be costly and time‐consuming for both the patient and the practitioners. Here, a short‐acting psychedelic such as 5‐MeO‐DMT would substantially lower treatment costs, resulting in a more financially accessible treatment option. As previously reviewed, the psychoactive effects of inhaled 5‐MeO‐DMT are immediate, reaching peak effects in a matter of seconds, and lasting up to 20 min.

The preliminary therapeutic evidence, as well as the potential pharmacological advantages of 5‐MeO‐DMT versus other classical psychedelics, has proven intriguing to researchers and pharmaceutical companies alike, fueling a growing number of clinical trials which are currently in various stages of development (Table 1). At present, two clinical studies have been registered at ClinicalTrials.gov with the aim to establish the safety of inhalable and intranasal formulations of 5‐MeO‐DMT in healthy volunteers. The inhalable formulation has also been administered to patients with treatment‐resistant depression in order to assess safety and efficacy. Both studies with the inhalable formulation have been completed and the safety data collected from healthy volunteers were recently published (Reckweg et al., 2021). This study aimed to assess the impact of four different dose levels of a novel vaporized 5‐MeO‐DMT formulation administered via inhalation as single doses of 2, 6, 12, and 18 mg and in an individualized dose escalation regimen on the safety, tolerability and the dose‐related psychoactive effects in healthy volunteers. Higher doses of 5‐MeO‐DMT produced short‐acting, significant increments in the intensity of the psychedelic experience ratings compared with the lowest 2 mg dose as assessed with a Peak Experience Scale (PES), the Mystical Experience Questionnaire (MEQ), the Ego Dissolution Inventory (EDI), and the 5‐Dimensional Altered States of Consciousness Questionnaire (5D‐ASC). Yet, maximal psychedelic experiences were observed following individualized dose escalation of 5‐MeO‐DMT of 6, 12, and 18 mg, where at least one and up to three doses of 5‐MeO‐DMT interspaced at 3 h were given on a single day. These data, therefore, suggest that individualized dose escalation of 5‐MeO‐DMT may be best suited for clinical applications that aim to maximize the experience to elicit a strong therapeutic response. Maximizing the 5‐MeO‐DMT experience in clinical populations is relevant because strong associations between psychedelic peak experiences and therapeutic effects have been reported for other tryptamines (Garcia‐Romeu et al., 2014; Johnson et al., 2014; Bogenschutz et al., 2015; Griffiths et al., 2016; Roseman et al., 2018; Ross et al., 2016).

On average, the duration of the psychedelic experience with 5‐MeO‐DMT was estimated to last 15–20 min. After the psychoactive effects have worn off, both cognitive and psychomotor function also quickly return to baseline. Likewise, vital signs at 1 and 3 h after administration were not affected and adverse events were generally mild and resolved spontaneously (Reckweg et al., 2021). As well as attesting to the clinical cost‐effectiveness of such a short‐lasting psychedelic experience, the rapid dissolution of drug‐induced cognitive and psychomotor dysfunction also suggests the safety of 5‐MeO‐DMT in relation to day‐to‐day operations requiring skilled performance, suggesting a less time‐intensive procedure for patients as well.

As such, the ability of 5‐MeO‐DMT to induce peak effects of similar intensity to high‐dose psilocybin (Barsuglia et al., 2018), but in such a short amount of time, is a facet of the 5‐MeO‐DMT experience that is of particular therapeutic interest. Additionally, as demonstrated by Reckweg and colleagues (Reckweg et al., 2021), if a peak experience is not achieved upon first administration, a dose‐escalation scheme can be safely employed in order to reliably induce such an experience. Thus there seems to be a very little build‐up of tolerance to the effects of 5‐MeO‐DMT, which is in contrast to other psychedelic substances (Nichols, 2016).

## 8 TOAD OR SYNTHETIC SOURCE OF 5‐MEO‐DMT

The main difference between toad venom of the *Bufo alvarius* and synthetic 5‐MeO‐DMT is that the former contains a range of pharmacologically active compounds in addition to 5‐MeO‐DMT. These include cardioactive agents such as bufagins (i.e., bufandienolides), catecholamines, such as epinephrine and norepinephrine, indolealkylamines, such as bufothionine, serotonin, cinobufotenine, bufotenine, and dehydrobufotenine, and noncardiac sterols, such as cholesterol, provitamin D, gamma sitosterol, and ergosterol (Erspamer et al., 1965; Erspamer et al., 1967; Chen & Kovarikova, 1967). It is presently unknown if any of these additional compounds synergistically modulate the effects of 5‐MeO‐DMT in ways that would be relevant for therapeutic applications. Although research into the “entourage” effects of toad venom would be of fundamental scientific interest, it is likely that the pharmacological constellation of toad venom is too complex and variable to be seriously considered as a target product for clinical drug development. Moreover, mental health improvements that have been associated with 5‐MeO‐DMT were similar for users of toad venom and synthetic versions of 5‐MeO‐DMT which suggest that 5‐MeO‐DMT is the primary compound with therapeutic potential (Uthaug, Lancelotta, Szabo, et al., 2020; Uthaug et al., 2019; Davis et al., 2019).

There are also ethical and ecological arguments not to pursue clinical development of toad venom (Uthaug, 2018). At present, the Sonoran Desert toad is not classified as endangered on the International Union for Conservation of Nature (IUCN) red list of threatened species, however, this classification dates back to an assessment of the toad population in 2004 (Hammerson & Santos‐Barrera, 2004). Thus, it is plausible that the increasing popularity of inhalation of 5‐MeO‐DMT vapor from dried toad secretion in under‐ground ceremonies may affect the stability of the toad population in the long run. Moreover, it is likely that the increasing demand for the 5‐MeO‐DMT toad secretion will disturb the ecological equilibrium of the toads through the invasion of habitat, excessive milking, amphibian trafficking, and black‐market dynamics (Wake & Vredenburg, 2008). The potential extinction of the Sonoran Desert Toad can be easily prevented by switching to synthetic forms of 5‐MeO‐DMT that in addition allow full control over their pharmacological constellation and hence, their safety of use.

## 9 DISCUSSION

5‐MeO‐DMT is a fast‐acting tryptamine that can induce an immediate (within seconds) and intense psychedelic experience of short duration (10–20 min). Core to the psychedelic experience is the feeling of ego dissolution, described as a sense of oneness with the universe or the experience of relaxed boundaries between the self and the world, in the absence of visual imagery. The intensity and duration of subjective effects produced by 5‐MeO‐DMT however may differ between routes of administration. 5‐MeO‐DMT has high binding affinities for the 5‐HT1A and 5‐HT2A receptors but appears to be more selective for 5‐HT1A. Adverse events associated with 5‐MeO‐DMT are mild and transient and can include anxiety, confusion, paranoia, loss of body perception, and flashbacks/reactivations. Observational studies and surveys have suggested that single administrations of 5‐MeO‐DMT, like other tryptamines, could be employed to induce durable treatment of mental health disorders such as depression, anxiety, PTSD, and substance misuse. Such disorders often coexist in individuals, which can limit treatment outcomes of conventional treatment approaches (Hirschfeld, 2001; Quello et al., 2005). A treatment that can target comorbid pathologies collectively could be expected to yield improved clinical outcomes. In contrast to other psychedelics, intervention with 5‐MeO‐DMT could be of short duration, which would reduce costs and time for both the patient and the practitioner. Additionally, a significant predictor of the therapeutic benefit of a psychedelic seems to be the peak experience (Davis et al., 2018; Roseman et al., 2018), a state in which 5‐MeO‐DMT appears to be very well suited to elicit (Reckweg et al., 2021).

Although clinical development of 5‐MeO‐DMT is in its infancy, there are a number of entities developing various formulations of the substance for clinical use (see Table 1). At the forefront, GH research is a publicly traded biotechnology company focusing on developing inhalable and injectable formulations of 5‐MeO‐DMT for psychiatric conditions (GHresearch, 2021). The aforementioned dose‐finding study with inhalable 5‐MeO‐DMT (Reckweg et al., 2021) was conducted in preparation for a clinical trial in patients with treatment‐resistant depression. That study has been completed and safety and efficacy data are expected to be published in the near future. Beckley Psytech, a clinical stage privately held company, has also initiated a phase I trial assessing the safety, tolerability, and pharmacokinetics of single ascending doses of a 5‐MeO‐DMT intranasal formulation (BeckleyPsytech 2021). Alongside the development of the drug, Beckley Psytech is also co‐developing a training program for therapists who will be assisting in future 5‐MeO‐DMT clinical trials (Carpenter, 2021). Other privately held companies have also declared their interest in developing the substance for various indications and in various formulations. Alvarius Pharmaceuticals is reportedly investigating 5‐MeO‐DMT for cocaine and heroin use disorder (AlvariusPharmaceuticals, 2021), CB therapeutics has claimed development of a formulation of 5‐MeO‐DMT via yeast strains (CBTherapeutics, 2021), CaaMTech has reported development of orally active 5‐MeO‐DMT analogs and compositions containing toad secretion tryptamines (CaaMTech, 2021), and Biomind Labs has reported the development of a thermosensitive nasal gel as an easily applicable formulation to administer 5‐MeO‐DMT (Businesswire, 2021) as an anti‐inflammatory (Biomind, 2021). Outside of the profit sector, USONA Institute, a nonprofit medical research organization dedicated to supporting and conducting research into the therapeutic potential of psychedelics (Usona, 2021), has published a detailed 5‐MeO‐DMT synthesis process (Sherwood et al., 2020) as well as guidelines for the assessment of the substances’ safety and efficacy in human clinical trials (Sherwood et al., 2019). The company’s commitment to open science can assist and streamline further clinical research and development with 5‐MeO‐DMT for future organizations.

An important issue regarding the therapeutic potential of 5‐MeO‐DMT is the lack of controlled, larger cohort clinical studies. Besides appropriate dosing, a standardized route of administration may also be critical with regard to the therapeutic outcome (Uthaug, Lancelotta, Szabo, et al., 2020; Uthaug et al., 2019; Sepeda et al., 2020). A variety of routes of administration such as vaporisation (Reckweg et al., 2021) and intranasal, intravenous, and IM administrations appear feasible to standardize. It is expected that clinical studies in the near future will incorporate these different routes of administration in their design to determine and compare their pharmacokinetic and pharmacodynamic profiles. Such studies should also consider the biological effects of 5‐MeO‐DMT including its modulatory potential on physiological parameters such as biomarkers of inflammation and neuroplasticity. Correlation analyses between pharmacokinetic data, routes of administration, biomarker levels, and psychotherapeutic potential will help to optimize clinical treatment modalities in patient populations, as well as to unveil the underlying bio‐psychological principles that contribute to healing. In a similar vein, harnessing the possible anti‐inflammatory effects of 5‐MeO‐DMT in for example, autoimmune disorders, and the potential contribution of psychological components of the session/therapy to these would require focused, larger cohort studies with correlational components and, ideally, a follow‐up design (Thompson & Szabo, 2020).

Observational research (Uthaug et al., 2019) has suggested that improvements in mental health outcomes following a single exposure to 5‐MeO‐DMT can sustain for months. The fundamental mechanism driving clinical change as well its durability over time however is currently unknown. A more fundamental, mechanistic understanding of psychological as well as biological processes that are activated during and following 5‐MeO‐DMT exposure will be seminal for the development of a 5‐MeO‐DMT treatment program that aims to achieve a rapid relief of psychopathological symptoms as well as control over a sustained response. Neurophysiological changes may be an important driver of psychological changes in mindset that are reported after the use of 5‐MeO‐DMT. It appears relevant to investigate whether 5‐MeO‐DMT induced changes in mindset can be conserved with psychological interventions that sustain individual beliefs that orient the way how day‐to‐day challenges and events are handled efficiently and productively. A sustained psychological state of well‐being may also feedback positively on neurophysiological biomarkers of psychopathological states and prevent relapse. In such a scenario, neurophysiological and psychological considerations of mental health changes observed after psychedelics do not have to be mutually exclusive and can offer complementary perspectives.

In sum, clinical research on the therapeutic potential of 5‐MeO‐DMT seems justified as preclinical, observational, and survey data provide a signal of its potential utility in mental health treatment. The rapid onset and short duration of the 5‐MeO‐DMT experience may render it more cost‐effective compared with longer‐acting psychedelics. A range of biotech companies has shown a particular interest in the production of 5‐MeO‐DMT formulations and drug development programs that can launch these formulations into clinical applications. Commercial development will therefore be the most important resource for bringing 5‐MeO‐DMT to the clinic in the near future. It should be noted however that fundamental research will be needed in addition in order to create an understanding of the neurophysiological and neural mechanisms that contribute to potential clinical effects of 5‐MeO‐DMT. Such studies are less likely to be conducted as part of drug development programs and will depend more on independent, academic initiatives.

## CONFLICTS OF INTEREST

JTR and JGR are scientific consultants to GH Research. MVU is a scientific consultant to Entheon. AKD is supported by private philanthropic funding from Tim Ferriss, Matt Mullenweg, Craig Nerenberg, Blake Mycoskie, and the Steven and Alexandra Cohen Foundation. AKD is also supported by the Center for Psychedelic Drug Research and Education, funded by anonymous private donors. The funding sources had no role in the study, data analysis, interpretation, or communication of findings.

## AUTHOR CONTRIBUTIONS

JTR, MVU, AS, AKD, RL, NLM, and JGR prepared the article and figures. All authors have approved the content of the article.

## Data Availability

Data sharing is not applicable to this article as no new data were created or analyzed in this review.
